# Nanomaterials Based on Fe_3_O_4_ and Phthalocyanines Derived from Cashew Nut Shell Liquid

**DOI:** 10.3390/molecules24183284

**Published:** 2019-09-09

**Authors:** Viviane G. P. Ribeiro, João P. F. Mota, Antônio E. Costa Júnior, Nayane M. A. Lima, Pierre B. A. Fechine, Juliano C. Denardin, Luigi Carbone, Ermelinda Bloise, Giuseppe Mele, Selma E. Mazzetto

**Affiliations:** 1 Laboratory of Products and Process Technology (LPT), Organic and Inorganic Chemistry Department, Federal University of Ceará (UFC), Campus do Pici, Fortaleza-CE 60440-900, Brazil; vivianegpribeiro@live.com (V.G.P.R.); jpfmpro@gmail.com (J.P.F.M.); eufraziojr@yahoo.com.br (A.E.C.J.); nayaneal@yahoo.com.br (N.M.A.L.); selma@ufc.br (S.E.M.); 2 Group of Chemistry of Advanced Materials (GQMat)—Department of Analytical Chemistry and Physical-Chemistry, Federal University of Ceará—UFC, Campus do Pici, CP 12100, Fortaleza, CE CEP 60451-970, Brazil; fechine@ufc.br; 3 Department of Physics, Universidad de Santiago de Chile and CEDENNA, USACH, Av. Ecuador, Santiago 3493, Chile; juliano.denardin@usach.cl; 4 CNR NANOTEC-Istituto di Nanotecnologia, c/o Campus Ecotekne, Università del Salento, Via Monteroni, 73100 Lecce, Italy; luigi.carbone@nanotec.cnr.it; 5 Department of Engineering for Innovation, University of Salento, Via Arnesano, 73100 Lecce, Italy; ermelinda.bloise@unisalento.it

**Keywords:** nanomaterials, cashew nut shell liquid (CNSL), phthalocyanines, superparamagnetic

## Abstract

In this work we report the synthesis of new hybrid nanomaterials in the core/shell/shell morphology, consisting of a magnetite core (Fe_3_O_4_) and two consecutive layers of oleic acid (OA) and phthalocyanine molecules, the latter derived from cashew nut shell liquid (CNSL). The synthesis of Fe_3_O_4_ nanoparticle was performed via co-precipitation procedure, followed by the nanoparticle coating with OA by hydrothermal method. The phthalocyanines anchorage on the Fe_3_O_4_/OA core/shell nanomaterial was performed by facile and effective sonication method. The as obtained Fe_3_O_4_/OA/phthalocyanine hybrids were investigated by Fourier transform infrared spectroscopy, X-ray diffraction, UV-visible spectroscopy, transmission electron microscopy (TEM), thermogravimetric analysis and magnetic measurements. TEM showed round-shaped nanomaterials with sizes in the range of 12–15 nm. Nanomaterials presented saturation magnetization (Ms) in the 1–16 emu/g and superparamagnetic behavior. Furthermore, it was observed that the thermal stability of the samples was directly affected by the insertion of different transition metals in the ring cavity of the phthalocyanine molecule.

## 1. Introduction

Nanomaterials containing Fe_3_O_4_ nanoparticles have received great attention due to their promising and multifunctional properties, besides potential medical applications in magnetic resonance imaging (MRI) and for cancer therapy [1], water treatment membranes [2], catalysis [3], magnetic separation [4] and detection DNA [5]. Previous studies [6] have shown the possibility of forming bifunctional therapeutic agents for use in photodynamic therapy (PDT) and magnetic hyperthermia. Specifically, the PDT aims at the treatment of several types of cancer, which combines a photosensitizer drug and light to generate active species of oxygen that fight the disease [7]. For this application, phthalocyanines commonly appear as excellent photosensitizers.

Phthalocyanines and their metal complexes have been considered versatile molecules, with remarkable optical and lasing properties, thermal and chemical stabilities [8], with maximum light absorption occurring in the visible region as well as being capable of producing singlet oxygen with high quantum yields [9]. Moreover, they have shown several extents of applicability in the fields of solar cells [10], liquid crystals [11] and as systems for fabrication of organic light-emitting diodes (OLED) [12]. Phthalocyanines consist of an aromatic macrocycle with an extensive conjugated π-electron system that can be readily modified by central coordination of metal atoms and peripheral substitution on the phthalocyanine ring, either by electron-donating or withdrawing groups [13,14].

The preparation of new eco-friendly fine chemicals derived from renewable and natural sources is of great interest. In this sense, the synthesis of cardanol-based phthalocyanines has been described in the literature [15,16,17]. Cardanol is the main component of technical cashew nut shell liquid (*t*-CNSL), obtained as a by-product during the industrial processing of cashew nut (*Anacardium occidentale* L.). *t*-CNSL is considered one of the richest natural sources of phenolic compounds; it is a renewable oil with low commercial value [18,19]. It consists of a mixture of long alkyl chain phenols (cardanol, cardol, anacardic acid and 2-methylcardol), with interesting properties and the possibility of chemical transformation for various applications [20,21,22,23].

Many studies related to hybrids have described the chemical interaction between phthalocyanine molecules and solid-state nanoparticles occurring through phthalocyanine’s peripheral functional groups that can anchor the particles’ surfaces [24,25,26,27]. Other reports have addressed a physical interaction, including alkyl chains in the phthalocyanine peripheral structure [28,29,30]. In the present study we report the successful synthesis of novel magnetic nanomaterials made by the combination of magnetite Fe_3_O_4_ nanoparticles, with the surface functionalized in sequence with oleic acid and cardanol-derived phthalocyanines; the latter passivation is favored by Van der Waals interactions between alkyl chains. We show also the influence of different metals coordinated to the center of phthalocyanines on the thermal and magnetic properties of these hybrid inorganic–organic nanomaterials.

## 2. Results

### 2.1. FT-IR Spectra

**Figure 1:**
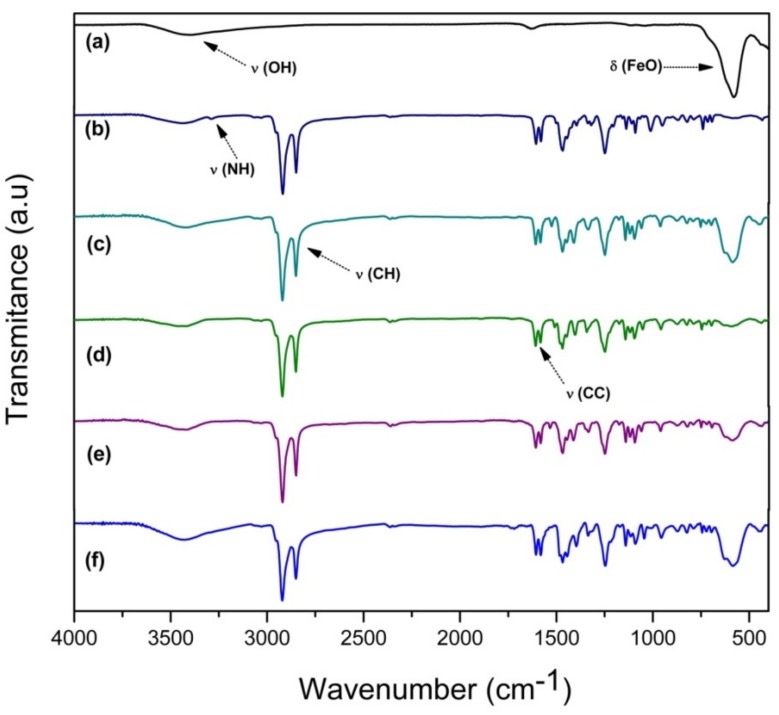
FT-IR analysis of (**a**) Fe_3_O_4_; (**b**) Fe_3_O_4_@OA/Pc; (**c**) Fe_3_O_4_@OA/CoPc; (**d**) Fe_3_O_4_@OA/CuPc; (**e**) Fe_3_O_4_@OA/NiPc; and (**f**) Fe_3_O_4_@OA/ZnPc.

FT-IR spectroscopy is usually employed for the fast and efficient identification of chemical molecules and, in this view, of molecular ligands possibly functionalizing magnetite nanoparticles [31]. FTIR carried out on Fe_3_O_4_ sample shows characteristic bands of Fe–O at 580 cm^−1^, as seen in Figure 1a. The presence of hydroxyl groups that reside on the Fe_3_O_4_ nanoparticles’ surface is attributed to the synthetic procedure conducted in aqueous media. The vibrational modes assigned to O–H stretching were observed at 3411cm^−1^ and the band at 1637cm^−1^ was attributed to angular vibration of O–H. As expected, the characteristic bands of Fe_3_O_4_ nanoparticles decreased after coating (Figure 1b–f). The spectrum of the Fe_3_O_4_@OA/Pc (Figure 1b) presents a band at 3292 cm^−1^ attributed to the N–H stretching of phthalocyanine core [32]. The spectra of other hybrids, based on different metal phthalocyanines (Fe_3_O_4_@OA/CoPc, Fe_3_O_4_@OA/CuPc, Fe_3_O_4_@OA/NiPc and Fe_3_O_4_@OA/ZnPc) are virtually identical; bands between 2950 and 2850 cm^−1^ were assigned to the C–H bond stretching from aliphatic groups, and the bands at 1610 cm^−1^ to the aromatic C=C bonds stretching. The absence of the characteristic absorption bands at 3292 and 1014 cm^−1^, assigned to N–H bond vibration, confirms that no free-base phthalocyanine is present.

### 2.2. X-Ray Powder Diffraction

**Figure 2:**
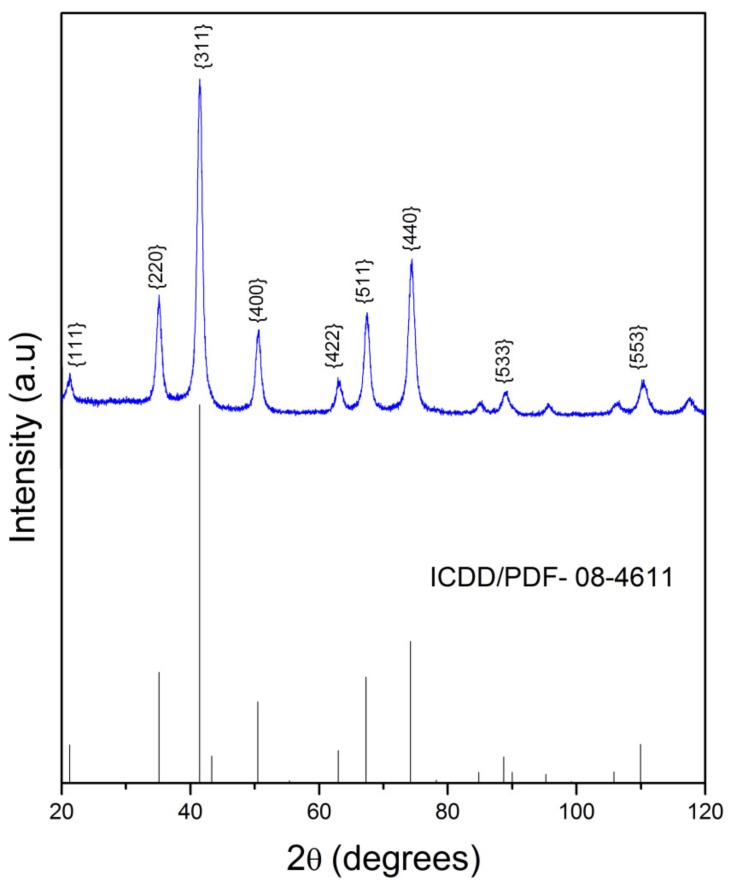
X-ray powder diffraction pattern of Fe_3_O_4_.

XRD analysis was performed to study the crystalline structure of the Fe_3_O_4_ nanoparticles. As shown in Figure 2 nine diffraction peaks were viewed at 2θ = 21.5°, 35.2°, 41.5°, 50.6°, 63.1°, 67.4°, 74.5°, 88.9° and 110.5°, corresponding to (hkl) Miller indices of (111), (220), (311), (400), (422), (511), (440), (533) and (553), respectively. All peaks were consistent with the reference pattern of the Fe_3_O_4_ cubic inverse spinel structure (JCPDS card # N° 08-4611) with spatial group Fd3M. The size of the Fe_3_O_4_ was calculated using the Debye–Scherrer equation (1.0) based on the (311) peak.

(1) $$D=\frac{K.\lambda }{\beta .cos\;\theta }$$

where *D* is crystal size (nm), *K* is a constant with value 0.9, *λ* is the wavelength of the X-ray radiation source (*λ* = 0.17889 nm, CoKα1), *β* is the measure of the width of maximum diffraction peak at the point where the intensity falls in half or “full width at half maximum (FWHM)”, and *θ* is the peak position [33]. The crystal size calculated for magnetite was of 11 nm, a value compatible with those already reported in the literature [34,35].

### 2.3. Thermal Analysis

**Figure 3:**
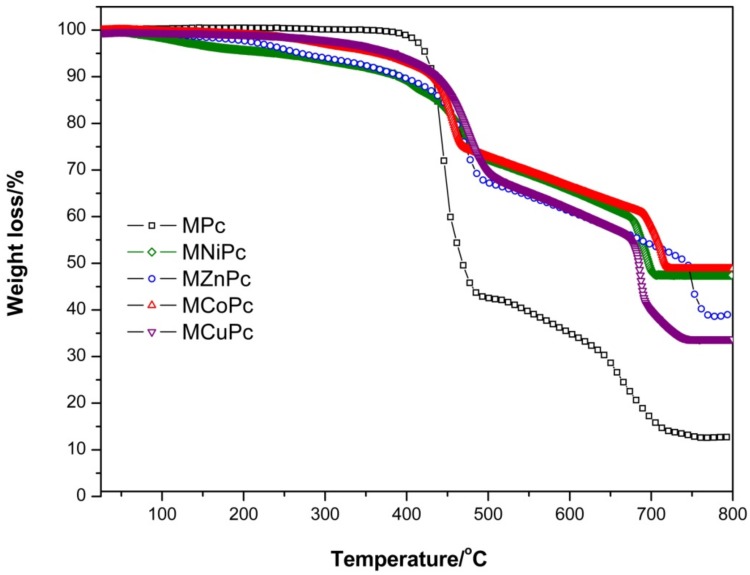
Thermogravimetric analysis of the Fe_3_O_4_@OA/Pcs under inert atmosphere.

Thermogravimetric results of the Fe_3_O_4_@OA/Pc, Fe_3_O_4_@OA/CoPc, Fe_3_O_4_@OA/CuPc, Fe_3_O_4_@OA/NiPc and Fe_3_O_4_@OA/ZnPc samples under a heating rate of 10 °C min^−1^, and N_2_ atmosphere, are shown in Table 1 and Figure 3. The decomposition of the materials occurred in two different stages and presented distinct residual masses in the range of 12–48%, which correspond to their metal oxides. All samples showed gradual weight loss of 34–65% in the temperature range of 167–740 °C. This event corresponds to the degradation of oleic acid and of peripheral chains attached to the macrocyclic moiety of the macromolecule. Decomposition and smaller weight losses of 13–22%, observed in the temperature range of 600–800 °C, correspond to the oxidative degradation of macrocyclic moiety of the phthalocyanines. Moreover, it was observed that there was a larger mass loss in Fe_3_O_4_@OA/Pc around 400 °C than in the other nanomaterials. This may be related to differences in the stacking arrangement of phthalocyanine molecules on the nanoparticle surface, which in the case of the free base forms aggregates, by π–π interaction, more efficiently than metallophthalocyanines when interacting with Fe_3_O_4_@OA, influencing the final mass of nanomaterial. In general, the complex aggregated with magnetite showed a initial degradation temperature lower than that of the non-metaled macromolecule. According to Barreto et. al. [34], this behavior is associated with the capacity of the nanoparticles to act as promoters of the degradation process. The order of observed thermal stabilities of metal complexes in inert atmosphere was: Fe_3_O_4_@OA/Pc > Fe_3_O_4_@OA/CuPc > Fe_3_O_4_@OA/CoPc > Fe_3_O_4_@OA/ZnPc > Fe_3_O_4_@OA/NiPc.

### 2.4. Transmission Electron Microscopy

**Figure 4:**
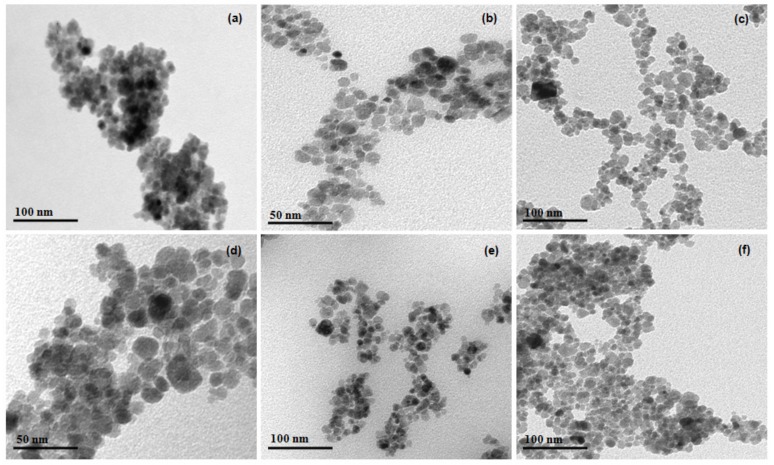
TEM images of (**a**) Fe_3_O_4_; (**b**) Fe_3_O_4_@OA/Pc; (**c**) Fe_3_O_4_@OA/CuPc; (**d**) Fe_3_O_4_@OA/NiPc; (**e**) Fe_3_O_4_@OA/ZnPc; and (**f**) Fe_3_O_4_@OA/CoPc.

The morphology and particle size of the samples were studied in more detail by TEM analysis, as shown in Figure 4. The TEM images showed that most of nanoparticles exhibited a roughly spherical shape and the spontaneous tendency to aggregate when deposited out from a solution onto a substrate. The Van der Waals and π-conjugated macrocyclic interactions of the phthalocyanines in Fe_3_O_4_ surface seem to favor this agglomeration. Moreover, it was possible to observe nanoparticle sizes with diameters of 12 ± 2 nm, 16 ± 3 nm, 13 ± 2 nm, 15 ± 4 nm and 13 ± 2 nm for Fe_3_O_4_@OA/Pc, Fe_3_O_4_@OA/CuPc, Fe_3_O_4_@OA/NiPc, Fe_3_O_4_@OA/ZnPc and Fe_3_O_4_@OA/CoPc, respectively. Fe_3_O_4_ isolated with a diameter of 10 ± 2 nm tended to agglomerate due to strong interparticle magneto-dipole interactions [35], but the coating with organic molecules noticeably decreased this behavior, as observed for the magnetic nanomaterials (Figure 4).

### 2.5. Magnetic Measurements

**Figure 5:**
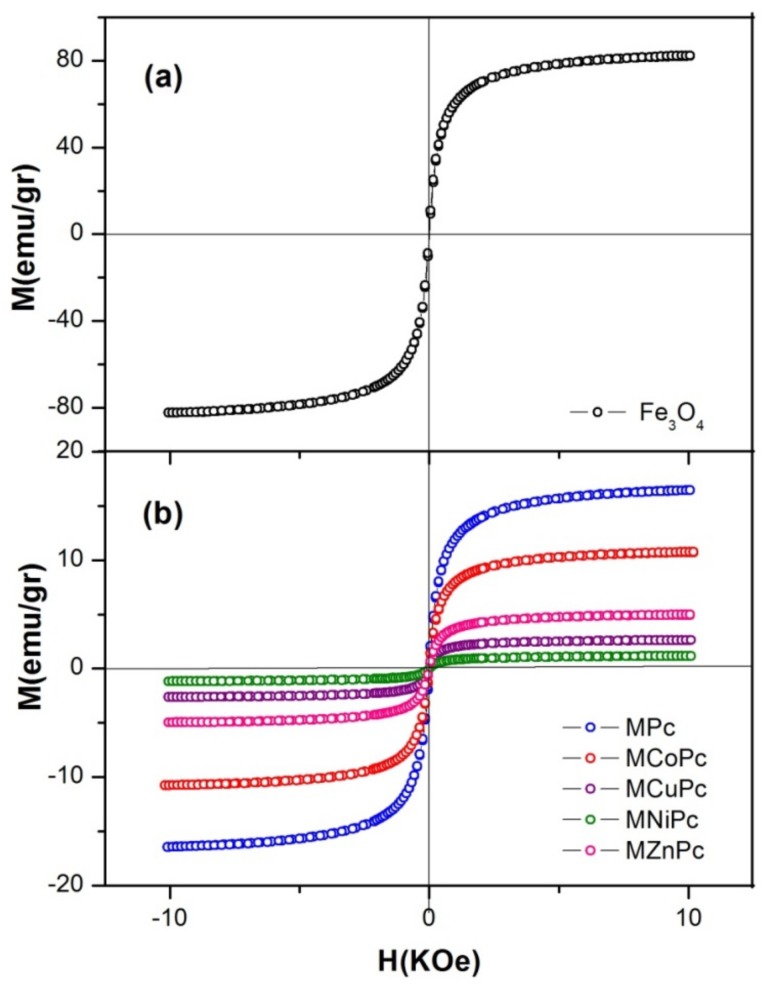
Magnetization curves of pure (**a**) Fe_3_O_4_ and (**b**) Fe_3_O_4_@OA/Pcs.

For verifying the influence of the different metals of the phthalocyanines on the magnetization of the nanomaterials, measures of the magnetization were performed as a function of magnetic field at room temperature using a vibrating sample magnetometer. The magnetization curves are presented in Figure 5. The magnetization curves of all nanomaterials exhibited a magnetic response to magnetic field variation and a characteristic superparamagnetic behavior, as evidenced in other previous works [17,36]. The values of saturation magnetization (Ms) found for Fe_3_O_4_@OA/Pc, Fe_3_O_4_@OA/CoPc, Fe_3_O_4_@OA/ZnPc, Fe_3_O_4_@OA/CuPc and Fe_3_O_4_@OA/NiPc were about 16, 11, 5, 3 and 1 emu/g, respectively. In general, the magnetization values obtained from the nanomaterials were significantly smaller than Fe_3_O_4_ nanoparticles (81 emu/g), in total agreement with similar examples reported in the literature [37]. At first such differences in magnetization values can be explained by the presence of non-magnetic components, namely oleic acid and phthalocyanines, which adhere to the nanoparticle surfaces, and by different relative Fe_3_O_4_ contents [36,37]. Thus, the reduction in saturation magnetization is basically due to the fact that these aggregated magnetite materials have a low magnetic response. Therefore, when phthalocyanine derivatives adhere to the nanoparticle surface, they contribute to the total sample mass, but do not contribute significantly with magnetic moments. Thus, like magnetization, the ratio of magnetic moments to mass (emu/g) showed a natural decreasing trend.

### 2.6. Optical Properties

**Figure 6:**
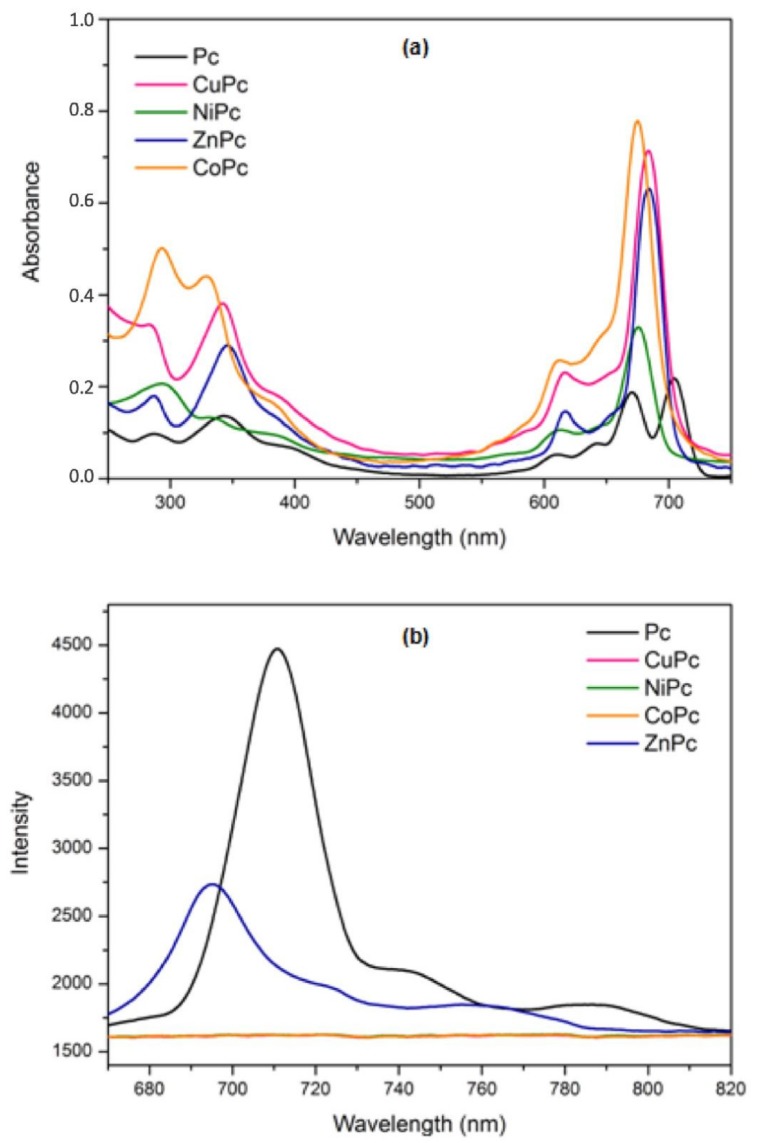
Absorption spectra (**a**) and emission spectra (**b**) of phthalocyanines.

The absorption and emission spectra of phthalocyanines in the ultraviolet-visible region are shown in Figure 6. Previous studies have shown that the presence of magnetic nanoparticles do not affect the absorbance and emission properties of the phthalocyanine derivatives [6]. Phthalocyanine exhibits typical electronic spectra with two absorption regions (Figure 6a), where the Q band is around 600–700 nm and the B band is near the UV region, around 300–450 nm. In metallo-phthalocyanines, Q bands consist of one sharp and intense band and two shoulders of low intensity, while in free base phthalocyanine these are of equal intensity. Figure 6b shows the maximum emission spectra of the phthalocyanines that occurs after excitation at 595 nm. The excitation spectra were similar to absorption spectra for Pc and ZnPc with similar wavelengths at 710 and 695 nm, respectively. This may suggest that the nuclear configurations of the ground and excited states are similar and not affected by excitation. This behavior is typical of phthalocyanine complexes [38]. The difference between the fluorescence intensities of the Pc and ZnPc was due to influence of axial metal coordinated to the center of macrocycle. It is important to note that emission corresponding in this spectral region was not observed for other compounds (NiPc, CoPc and CuPc), due to paramagnetic behavior of central metals in the phthalocyanine cavity [39]. 

## 3. Materials and Methods

### 3.1. Synthesis and Metallation of Phthalocyanines from CNSL

**Figure 7:**
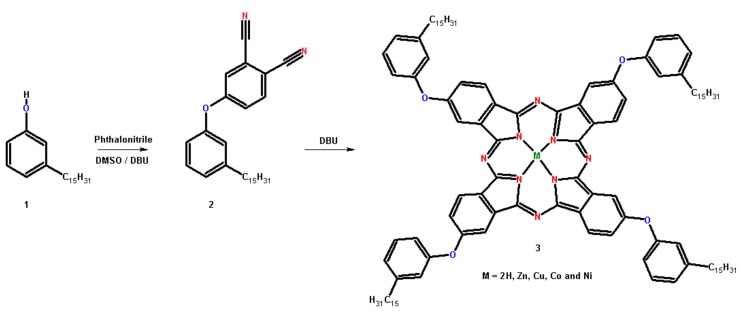
Synthetic route for phthalocyanines. (**1**) 3-pentadecylphenol; (**2**) 4-(3-pentadecylphenoxy)phthalonitrile; and (**3**) tetrakis-(3-pentadecylphenoxy) phthalocyanine.

Figure 7 shows the synthetic route of phthalocyanines obtained from hydrogenated cardanol; that is 3-*n*-pentadecylphenol (**1**). The compound (**1**) was preferred as starting material due to its homogeneous chemical composition [40]. The compounds 4-(3-pentadecylphenoxy)phthalonitrile (**2**) and tetrakis-(3-pentadecylphenoxy)phthalocyanine (**3**) were prepared according to procedures previously described in the literature [15,16,41] with some modifications. Metal-free phthalocyanine (**3**) was prepared by mixing 0.20 g of compound **2** and 1,8-diazabicyclo[5.4.0]undec-7-ene (DBU) (0.70 g, 70.0 µL) that was refluxed in butan-1-ol (10 mL) for 16 h. To obtain metallo-phthalocyanines 0.04 g of an acetate-based metal salt X(CH_3_COO)_2_2H_2_O (X = Zn, Cu, Co and Ni) was added to the reaction batch. After cooling to room temperature, ethanol was added and the solid filtered. The product was purified by column chromatography (silica gel) using methylene chloride (CH_2_Cl_2_) as eluent. The solvent was evaporated and the product obtained as a blue solid (**3**). Metal-free phthalocyanine (Pc): Yield 46%. ^1^H NMR (CDCl_3_, ppm) broad signals: −3.65 (2H), 0.84 (t, *J* = 8.4 Hz, 12H), 1.16–1.38 (m, 96H), 1.76 (m, 8H), 2.76 (t, *J* = 7.8 Hz, 8H), 7.13–8.62 (m, 28H). FT-IR (KBr, cm^−1^): 3292, 2922, 2852, 1606, 1583, 1523, 1467, 1249, 1140, 1093, 1012, 742. UV-vis (λmax/nm, CHCl_3_): 607, 639, 670, 704. LC-MS (m/z): 1724 [M + H]^–^. Cobalt phthalocyanine (CoPc): Yield 68%. ^1^H NMR (CDCl_3_, ppm): 0.84 (t, *J* = 6.9 Hz, 12H), 1.25 (s, 96H), 1.59 (m, 8H), 2.62 (t, *J* = 7.7 Hz, 8H), 7.70–6.83 (28H). FT-IR (KBr, cm^−1^): 2922, 2852, 1608, 1583, 1468, 1248, 1142, 960, 750. UV-vis (λmax/nm, CHCl_3_): 612, 675. Zinc phthalocyanine (ZnPc): Yield 55%. ^1^H NMR (CDCl_3_, ppm) broad signals: 0.94–0.86 (m, 12H), 1.65–1.08 (m, 104H), 2.62 (t, *J* = 7.8 Hz, 8H), 6.81–7.73 (28H). FT-IR (KBr, cm^−1^): 2922, 2852, 1606, 1581, 1485, 1246, 1142, 948, 744. UV-vis (λmax/nm, CHCl_3_): 616, 684. Nickel phthalocyanine (NiPc): Yield 63%. ^1^H NMR (CDCl_3_, ppm) broad signals: 0.84 (t, *J* = 6.5 Hz, 12H), 1.17–1.27 (m, 96H), 1.74 (m, 8H), 2.72 (d, *J* = 7.5 Hz, 8H), 7.08–7.76 (m, 28H). FT-IR (KBr, cm^-1^): 2923, 2852, 1608, 1583, 1473, 1250, 1143, 961, 751. UV-vis (λmax/nm, CHCl_3_): 612, 676. Copper phthalocyanine (CuPc): Yield 72%. ^1^H NMR (CDCl_3_, ppm) broad signals: 0.87 (d, J = 6.7 Hz, 12H), 1.26 (s, 96H), 1.61 (m, 8H), 2.63 (t, *J* = 8.6 Hz, 8H), 6.88–7.72 (28H). FT-IR (KBr, cm^−1^): 2921, 2852, 1607, 1582, 1467, 1250, 1143, 957, 746. UV-vis (λmax/nm, CHCl_3_): 617, 684. 

^13^C NMR (300 MHz, CDCl_3_), ^1^H NMR (300 MHz, CDCl_3_) and Mass Spectra (MALDI-TOF-MS) of the compounds (a) Pc, (b) CoPc, (c) CuPc, (d) NiPc and (e) ZnPc have been reported in the Supplementary Materials section respectively in Figure S1, Figure S2 and Figure S3. 

### 3.2. Synthesis of Magnetite

To form the spinel phase Fe_3_O_4_ the co-precipitation processing route was followed [34,35]. Thus, a solution of metal salts containing Fe^2+^/Fe^3+^ in the molar ratio of 1:2 was dissolved in Milli-Q water and heated to 80 °C. Then, under to vigorous stirring, a 30 wt% NH_4_OH solution was added until the mixture reached pH = 10. The as-obtained black precipitate was washed several times with Milli-Q water until the residual solution became neutral. Lastly, the magnetic nanoparticles were dried. The chemical reaction of Fe_3_O_4_ formation is written in Equation (2).

Fe^2+^ + 2Fe^3+^ + 8O^−^ → Fe_3_O_4_ + 4H_2_O (2)

### 3.3. Preparation of Magnetic Nanomaterials

**Figure 8:**
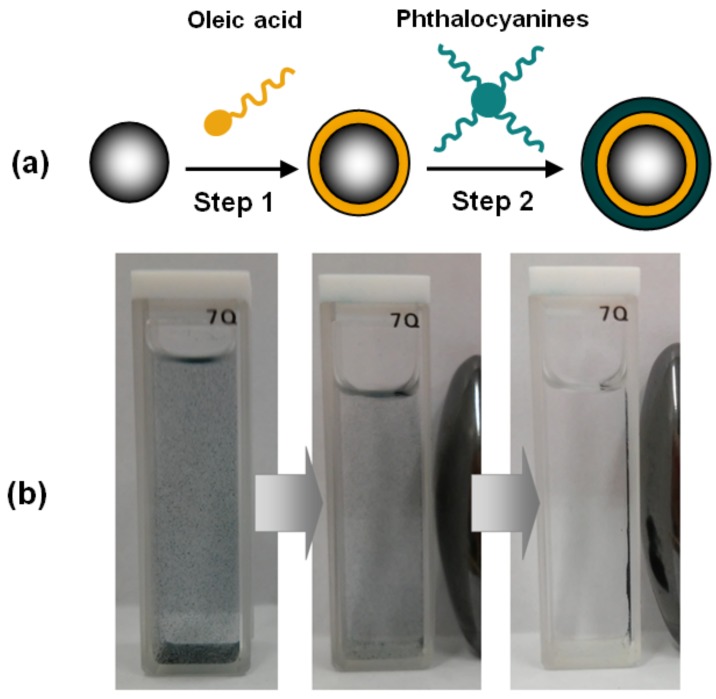
(**a**) Schematic representation of preparation of magnetic phthalocyanines: Step 1) ferrofluid produced with the mixture of Fe_3_O_4_ and oleic acid; Step 2) reaction of ferrofluid with phthalocyanines. (**b**) Influence of the magnetic field under the suspension of Fe_3_O_4_@OA/Pc in ethanol.

The hybrid nanomaterial consisted of magnetic nanoparticles stabilized by an inner layer of oleic acid, and an outer layer of phthalocyanine. Initially, magnetite (125.0 mg) was subjected to mechanical stirring in 50 mL of Milli-Q water, then few drops of NH_4_OH (30%) were added until pH = 10 was reached, afterwards, 50 mL of oleic acid (OA) were further incorporated in the batch. Posteriorly, the ferrofluid obtained was separated from the organic phase by magnetic attraction. The formation of the second passivation layer was performed through the addition of a solution of phthalocyanine (12.0 mg), dichloromethane (40 mL) and ethanol (60 mL) under sonication at 40 ºC for 30 min, with subsequent addition of an excess of ferrofluid (Figure 8a). The magnetic hybrid (Fe_3_O_4_@OA/Pc) was separated by using a magnet (Figure 8b). This methodology was reproduced for the preparation of all nanomaterials described herein (Fe_3_O_4_@OA/CoPc, Fe_3_O_4_@OA/NiPc, Fe_3_O_4_@OA/CuPc and Fe_3_O_4_@OA/ZnPc).

### 3.4. Characterization

FTIR measurements of the samples were performed using a Perkin Elmer 2000 spectrophotometer in a 400–4000 cm^−1^ range (KBr pellet). The phthalocyanines were analyzed by ^1^H NMR spectra recorded on a Bruker Avance DRX-500 (500 MHz ^1^H) using CDCl_3_ as solvent and by liquid chromatography–mass spectrometry (LC-MS) on a Bruker Microflex LT (Maldi Tof) using alphacyano and dichloromethane as a matrix, and 5% TFA as mobile phase. Furthermore, UV–Vis absorption was recorded by a Varian Cary 5000 spectrophotometer (in CHCl_3_). Photoluminescence excitation (PLE) measurements were obtained using a Horiba Nanolog fluorometer equipped with a liquid N_2_-cooled InGaAs detector with the excitation range from 410 to 550 nm (coming from a Xe lamp) and emission range from 600 to 800 nm. The X-ray powder diffraction (XRPD) analysis was performed in an X-ray powder diffractometer Xpert Pro MPD (Panalytical) using Bragg–Brentano geometry in the range of 20–120° with a rate of 1° min^−1^. CoKα radiation (λ = 1.7889 Å) was used and the tube operated at 40 kV and 30 mA, with the experimental patterns using the Rietveld algorithm to better identify and quantify crystallographic phases. Thermogravimetric analysis (TGA) experiments of the nanomaterials were all operated on a Mettler Toledo TGA/SDTA 851e equipment, under nitrogen atmospheres (constant flow of 50 cm^3^ min^−1^), with a heating rate of 10 °C min^-1^, sample mass of 10 mg, and temperature range from 25 to 800 °C. The magnetization measurements were performed at room temperature with a home-made vibrating sample magnetometer (VSM), calibrated using a pure Ni wire and, after measuring the mass of each sample, the magnetization was given in emu/g. The morphologies and microstructures of prepared samples were analyzed by a Jeol JEM-1011 transmission electron microscope (TEM) operating at 100 kV, equipped with a CCD camera ORIUS 831 from Gatan.

## 4. Conclusions

In summary, we demonstrated a facile strategy for the development of magnetic nanomaterials based on Fe_3_O_4_ and phthalocyanines derived from biomass. The results of TEM confirmed the nano-sized particles with spherical morphology. The decomposition temperatures of Fe_3_O_4_@OA/Pcs can reach 380 °C, indicating their excellent thermal stability. We also observed that, despite showing superparamagnetic behavior, the transition metals of metallo-phthalocyanines appear to negatively influence the magnitude of the nanomaterial magnetization. Thus, we showed that by Van der Waals interactions occurring between the phthalocyanines and oleic acid alkyl chains emerging from the surface of Fe_3_O_4_, it was possible to obtain stable and magnetic hybrid nanomaterials.

## Figures and Tables

**Table 1:** Degradation temperatures, mass losses and mass residues of pyrolysis decomposition at heating rates of 10 °C min^−1.^

Nanomaterials	First Stage		Second Stage		Residue/%
	Range/°C	Mass Loss/%	Range/°C	Mass Loss/%	
Fe_3_O_4_@OA/Pc	386–603	65.00	615–800	22.22	12.32
Fe_3_O_4_@OA/CoPc	292–673	35.39	675–641	12.88	48.71
Fe_3_O_4_@OA/CuPc	285–690	42.10	692–725	22.18	34.32
Fe_3_O_4_@OA/NiPc	255–673	34.71	675–707	12.53	47.95
Fe_3_O_4_@OA/ZnPc	167–740	47.74	741–784	12.07	38.83

## Supplementary Materials

The following are available online at https://www.mdpi.com/1420-3049/24/18/3284/s1, Figure S1: ^13^C NMR (300 MHz, CDCl_3_) of the (a) Pc, (b) CoPc, (c) CuPc, (d) NiPc and (e) ZnPc, Figure S2: ^1^H NMR (300 MHz, CDCl_3_) of the (a) Pc, (b) CoPc, (c) CuPc, (d) NiPc and (e) ZnPc, Figure S3: Mass Spectra (MALDI-TOF-MS) of the (a) Pc, (b) CuPc, (c) NiPc, (d) ZnPc and (e) CoPc.

## References

[1] Singh A.K., Srivastava O.N., Singh K. (2017). Shape and size-dependent magnetic properties of Fe_3_O_4_ nanoparticles synthesized using piperidine. Nanoscale Res. Lett..

[2] Ghaemi N., Madaeni S.S., Daraei P., Rajabi H., Zinadini S., Alizadeh R., Heydari R., Beygzadeh M., Ghouzivand S. (2015). Polyethersulfone membrane enhanced with iron oxide nanoparticles for copper removal from water: Application of new functionalized Fe_3_O_4_ nanoparticles. Chem. Eng. J..

[3] Kharisov B.I., Kharissova O.V., Rasika Dias H.V., Méndez U.O., La Fuente I.G., Peña Y., Dimas A.V., Luis N. (2016). Iron-based nanomaterials in the catalysis. Advanced Catalytic Materials—Photocatalysis and Other Current Trends.

[4] Dukenbayev K., Korolkov I.V., Tishkevich D.I., Kozlovskiy A.L., Trukhanov S.V., Gorin Y.G., Shumskaya E.E., Kaniukov E.Y., Vinnik D.A., Zdorovets M.V. (2019). Fe_3_O_4_ nanoparticles for complex targeted delivery and boron neutron capture therapy. Nanomaterials.

[5] Zhang J., Joshi P., Zhou Y., Ding R., Zhang P. (2015). Quantitative SERS-based DNA detection assisted by magnetic microspheres. Chem. Commun..

[6] Nyokong T., Antunes E. (2013). Influence of nanoparticle materials on the photophysical behavior of phthalocyanines. Coord. Chem. Rev..

[7] Er O., Colak S.G., Ocakoglu K., Ince M., Bresolí-Obach R., Mora M., Sagristá M.L., Yurt F., Nonell S. (2018). Selective photokilling of human pancreatic cancer cells using cetuximab-targeted mesoporous silica nanoparticles for delivery of zinc phthalocyanine. Molecules.

[8] Qian W., Wei W., Hong M., Jianfeng C., Guangwen C., Haikui Z. (2014). Microwave assisted synthesis of ZnPc-COOH and SiO_2_/ZnPc-COOH nanopaticles: Singlet oxygen production and photocatalytic property. Colloids Surf. A Physicochem. Eng. Aspects.

[9] Modisha P., Nyokong T. (2014). Fabrication of phthalocyanine-magnetic nanoparticles hybrid nanofibers for degradation of Orange-G. J. Mol. Catal. A Chem..

[10] Gomis L.M., Lázaro F.F., Santos A.S. (2014). Advances in phthalocyanine-sensitized solar cells (PcSSCs). J. Mater. Chem. A.

[11] Basova T.V., Parkhomenko R.G., Igumenov I.K., Hassan A., Durmus M., Gürek A.G., Ahsen V. (2014). Composites of liquid crystalline nickel phthalocyanine with gold nanoparticles: Liquid crystalline behaviour and optical properties. Dyes Pigments.

[12] Ma Y.Y., Hua X.C., Zhai T.S., Li Y.H., Lu X., Duhm S., Fung M.K. (2019). Doped copper phthalocyanine via an aqueous solution process for high-performance organic light-emitting diodes. Org. Electron..

[13] Özcesmeci I., Gelir A., Gül A. (2014). Synthesis and photophysical properties of indium (III) phthalocyanine derivatives. J. Lumin..

[14] Awaji A.I., Köksoy B., Durmuş M., Aljuhani A., Alraqa S.Y. (2019). Novel hexadeca-substituted metal free and zinc(II) phthalocyanines; Design, synthesis and photophysicochemical properties. Molecules.

[15] Attanasi O.A., Ciccarella G., Filippone P., Mele G., Spadavecchia J., Vasapollo G. (2003). Novel phthalocyanines containing cardanol derivatives. J. Porphy. Phthalocyanines.

[16] Slota R., Dyrda G., Hofer M., Mele G., Bloise E., Del Sole R. (2012). Novel lipophilic lanthanide bis-phthalocyanines functionalized by pentadecylphenoxy groups: Synthesis, characterization and UV-photostability. Molecules.

[17] Costa Junior A.E., Mota J.P.F., Pontes S.M.A., Maia F.J.N., Clemente C.S., Fechine P.B.A., Bohn F., Sales A.J.M., Sombra A.S.B., Carbone L. (2017). A self-assembly of graphene oxide@Fe_3_O_4_/metallo-phthalocyanine nanohybrid materials: Synthesis, characterization, dielectric and thermal properties. J. Mater. Sci..

[18] Ribeiro V.G.P., Marcelo A.M.P., da Silva K.T., da Silva F.L.F., Mota J.P.F., do Nascimento J.P.C., Sombra A.S.B., da Silva Clemente C., Mele G., Carbone L. (2017). New ZnO@Cardanol porphyrin composite nanomaterials with enhanced photocatalytic aapability under solar light irradiation. Materials.

[19] Lima N.M.A., Avila H.J.C., Marchiori C.F.N., Sampaio S.G., Mota J.P.F., Ribeiro V.G.P., Clemente C.S., Mele G., Cremona M., Mazzetto S.E. (2019). Light-emitting porphyrin derivative obtained from a subproduct of the cashew nut shell liquid: A promising material for OLED applications. Materials.

[20] Mota J.P.F., Ribeiro V.G.P., da Silva F.L.F., Costa Junior A.E., Oliveira D.R., Kotzebue L.R.V., Mele G., Lomonaco D., Mazzetto S.E. (2016). Developing eco-friendly methods for purification of compounds derived from hydrogenated cardanol. Sep. Sci. Technol..

[21] Lomonaco D., Mele G., Mazzetto S.E., Anilkumar P. (2017). Cashew Nut Shell Liquid (CNSL): Form an agro-industrial waste to a sustainable alternative to petrochemical resources. Cashew Nut Shell Liquid.

[22] Mota J.P.F., Costa Junior A.E., Ribeiro V.G.P., Sampaio S.G., Lima N.M.A., Clemente C.S., Mele G., Lomonaco D., Mazzetto S.E. (2016). Synthesis, characterization and dielectric properties of new 5-(4-hydroxyphenyl)-10,15,20-tri-4-[2-(3-pentadecylphenoxy)ethoxy]phenyl porphyrin and their Ni, Co and Cu complexes. J. Braz. Chem. Soc..

[23] Mele G., Lomonaco D., Mazzetto S.E., Anilkumar P. (2017). Cardanol-based heterocycles: Synthesis and applications. Cashew Nut Shell Liquid.

[24] Yan L., Pu Z., Xu M., Wei R., Liu X. (2017). Fabrication and Electromagnetic Properties of Conjugated NH_2_-CuPc@Fe_3_O_4_. J. Electron. Mater..

[25] Liu S., Liu C., Liu C., Tu L., You Y., Wei R., Liu X. (2019). Polyarylene ether nitrile and barium titanate nanocomposite plasticized by carboxylated zinc phthalocyanine buffer. Polymers.

[26] Matlou G.G., Oluwole D.O., Prinsloo E., Nyokong T. (2018). Photodynamic therapy activity of zinc phthalocyanine linked to folic acidand magnetic nanoparticles. J. Photochem. Photobiol. B Biol..

[27] Li K., Zheng P., Tong L., Ren D., Xu M., Tang X., Liu X. (2019). Design and properties of Poly(arylene ether nitriles) composites via incorporation of Poly(arylene ether nitriles) grafted Fe_3_O_4_/ Fe-phthalocyanine hybrid submicron-spheres. Compos. Part. B.

[28] Meng F., Zhao R., Zhan Y., Lei Y., Zhong J., Liu X. (2011). One-step synthesis of Fe-phthalocyanine/Fe3O4 hybrid microspheres. Mater. Lett..

[29] Karimi A.R., Azadikhah F., Rahimi L., Ghadimi S. (2015). Fabrication of new Fe-phthalocyanine oligomer–magnetite hybrid magnetic nano particles and their effects on the LCST behavior of thermo-sensitive poly(*N*-isopropylacrylamide-co-acrylic acid) magnetic nanocomposites. Colloids Surf. A Physicochem. Eng. Aspects.

[30] Li K., Ren D., Tang X., Xu M., Liu X. (2018). Micro/mesoporous Fe_3_O_4_/Fe-phthalocyanine microspheres and effects of their surface morphology on the crystallization and properties of poly(arylene ether nitrile) composites. Materials.

[31] Kumari A., Yadav S.K., Pakade Y.B., Singh B., Yadav S.C. (2010). Development of biodegradable nanoparticles for delivery of quercetin. Colloids Surf. B Biointerfaces.

[32] Bayrak R., Dumludag F., Akçay H.T., Degirmencioglu I. (2013). Synthesis, characterization and electrical properties of peripherally tetra-aldazine substituted novel metal free phthalocyanine and its zinc (II) and nickel (II) complexes. Spectrochim. Acta Part. A Mol. Biomol. Spectrosc..

[33] Modisha P., Nyokong T., Antunes E. (2013). Photodegradation of Orange-G using zinc octacarboxyphthalocyanine supported on Fe_3_O_4_ nanoparticles. J. Mol. Catal. A Chem..

[34] Barreto A.C.H., Maia F.J.N., Santiago V.R., Ribeiro V.G.P., Denardin J.C., Mele G., Carbone L., Lomonaco D., Mazzetto S.E., Fechine P.B.A. (2012). Novel ferrofluids coated with a renewable material obtained from cashew nut shell liquid. MicrofluidNanofluid.

[35] Ribeiro V.G.P., Barreto A.C.H., Denardin J.C., Mele G., Carbone L., Mazzetto S.E., Sousa E.M.B., Fechine P.B.A. (2013). Magnetic nanoparticles coated with anacardic acid derived from cashew nut shell liquid. J. Mater. Sci..

[36] Souza N.D.G., Freire R.M., Cunha A.P., da Silva M.A.S., Mazzetto S.E., Sombra A.S.B., Denardin J.C., Ricardo N.M.P.S., Fechine P.B.A. (2015). New magnetic nanobiocomposite based in galactomannan/glycerol and superparamagnetic nanoparticles. Mater. Chem. Phys..

[37] Li J., Wei J., Pu Z., Xu M., Jia K., Liu X. (2016). Influence of Fe_3_O_4_/Fe-phthalocyanine decorated graphene oxide on the microwave absorbing performance. J. Magn. Magn. Mater..

[38] Sevim A.M., Yenilmez H.Y., Bayir Z.A. (2013). Synthesis and photophysical properties of novel (trifluoromethyl)phenylethynyl-substituted metallophthalocyanines. Polyhedron.

[39] Kaya E.C., Durmus M., Yanmaz E., Kantekin H. (2014). Synthesis and spectral and thermal characterization of new metal-free and metallophthalocyanines: Investigation of their photophysical, photochemical, and thin film properties. Turk. J. Chem..

[40] Mele G., Vasapollo G. (2008). Fine chemicals and new hybrid materials from cardanol. Mini Rev. Org. Chem..

[41] Dilber G., Durmus M., Kantekin H., Çakir V. (2011). Synthesis and characterization of a new soluble metal-free and metallophthalocyanines bearing biphenyl-4-yl methoxy groups. J. Org. Chem..

