# Supplementary material for: Nanomaterials Based on Fe3O4 and Phthalocyanines Derived from Cashew Nut Shell Liquid
Source: Molecules. 2019 Sep 9;24(18):3284. doi: 10.3390/molecules24183284 (PMC6767058; doi:10.3390/molecules24183284)
Supplement: Supplementary file 1 [file molecules-24-03284-s001.pdf]

Supporting information

Nanomaterials based on Fe_3O_4 and phthalocyanines derived from cashew nut shell liquid

Viviane G. P. Ribeiro [1], João P. F. Mota [1], Antônio E. Costa Júnior [1], Nayane M. A. Lima [1], Pierre B. A. Fechine [2], Juliano C. Denardin [3], Luigi Carbone [4], Ermelinda Bloise [5], Giuseppe Mele [5]* and Selma E. Mazzetto [1]

[1] Laboratory of Products and Process Technology (LPT), Organic and Inorganic Chemistry Department, Federal University of Ceará (UFC), Campus do Pici, 60440-900, Fortaleza-CE, Brazil; vivianegpribeiro@live.com (V.R.); jpfmpro@gmail.com (J.P.); eufraziojr@yahoo.com.br (A.E); nayaneal@yahoo.com.br (N. L.); selma@ufc.br (S.M.)

[2] Group of Chemistry of Advanced Materials (GQMat) – Department of Analytical Chemistry and Physical-Chemistry, Federal University of Ceará – UFC, Campus do Pici, CP 12100, CEP 60451-970 Fortaleza, CE, Brazil; fechine@ufc.br (P.F.)

[3] Department of Physics, Universidad de Santiago de Chile and CEDENNA, USACH, Av. Ecuador, 3493 Santiago, Chile; juliano.denardin@usach.cl (J.D.)

[4] CNR NANOTEC-Istituto di Nanotecnologia, c/o Campus Ecotekne, Università del Salento, Via Monteroni, 73100 Lecce, Italy; *luigi.carbone*@nanotec.*cnr*.it (L.C.)

[5] Department of Engineering for Innovation, University of Salento, Via Arnesano, 73100, Lecce, Italy; ermelinda.bloise@unisalento.it (E.B.); giuseppe.mele@unisalento.it (G.M.)

* Correspondence: giuseppe.mele@unisalento.it

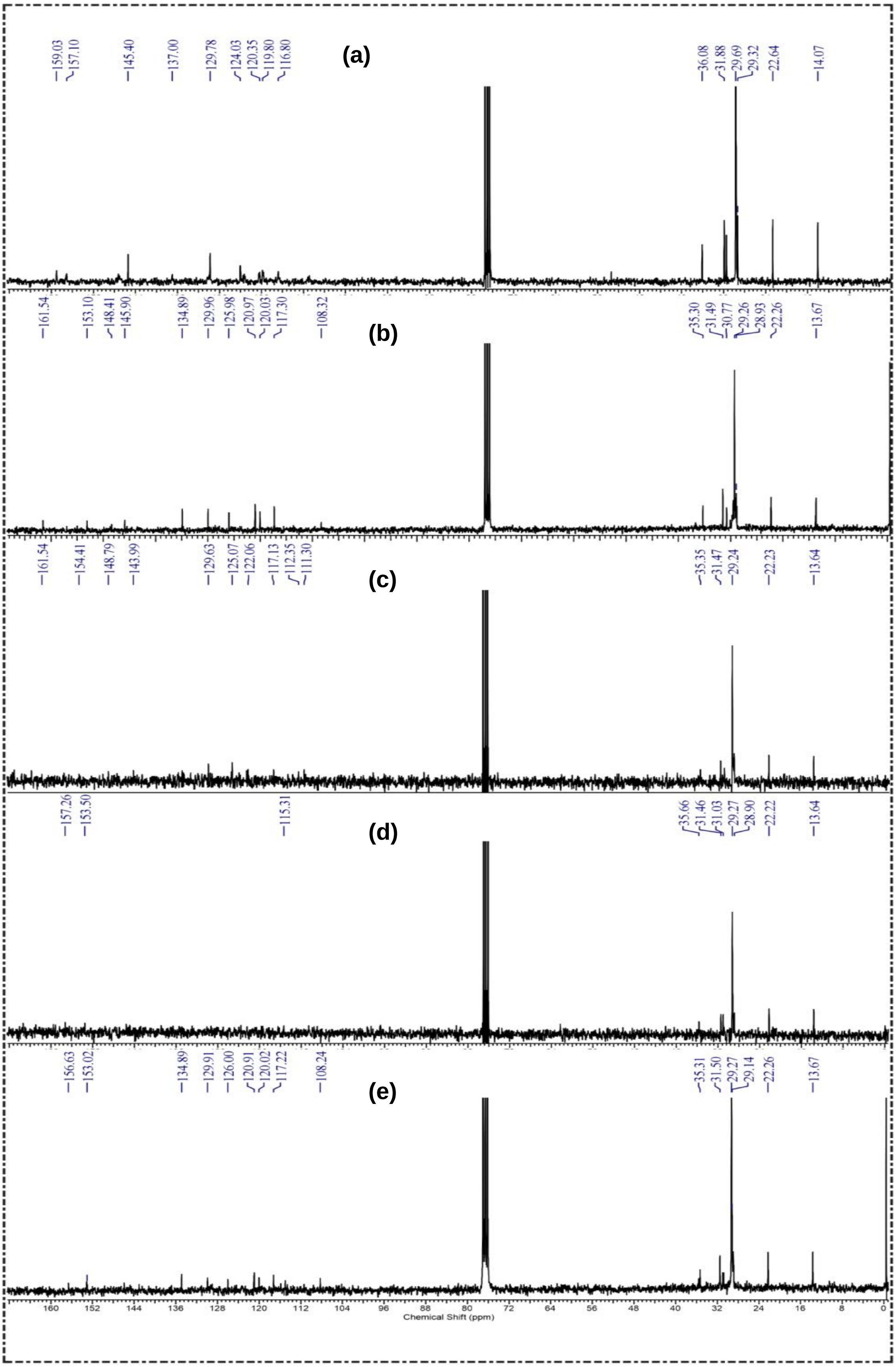

Figure S2. ^{1}H NMR (300 MHz, $CDCl_3$) of the (a) Pc, (b) CoPc, (c) CuPc, (d) NiPc and (e) ZnPc.

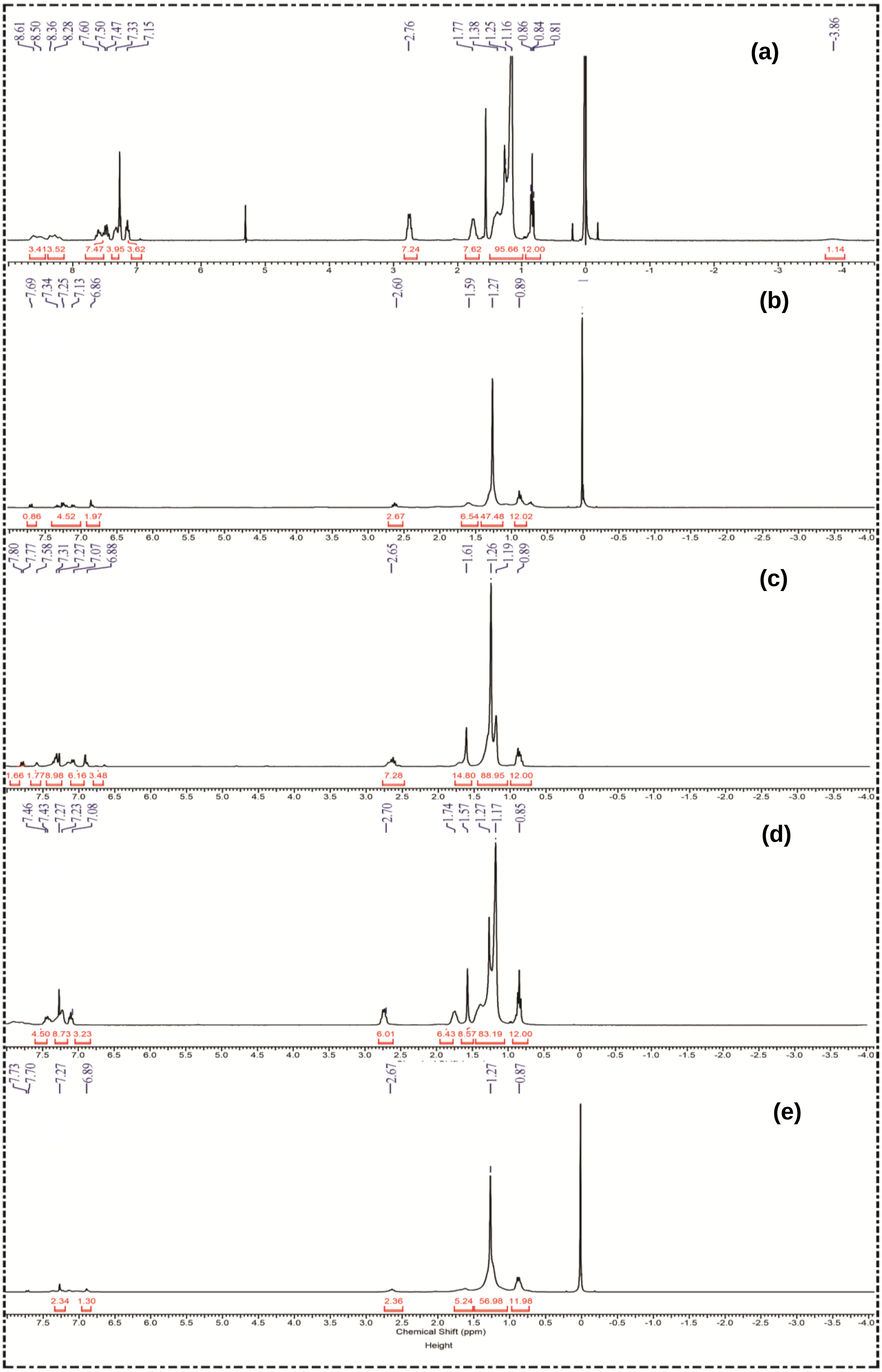

Figure S3. Mass Spectra (MALDI-TOF-MS) of the (a) Pc, (b) CuPc, (c) NiPc, (d) ZnPc and (e) CoPc.

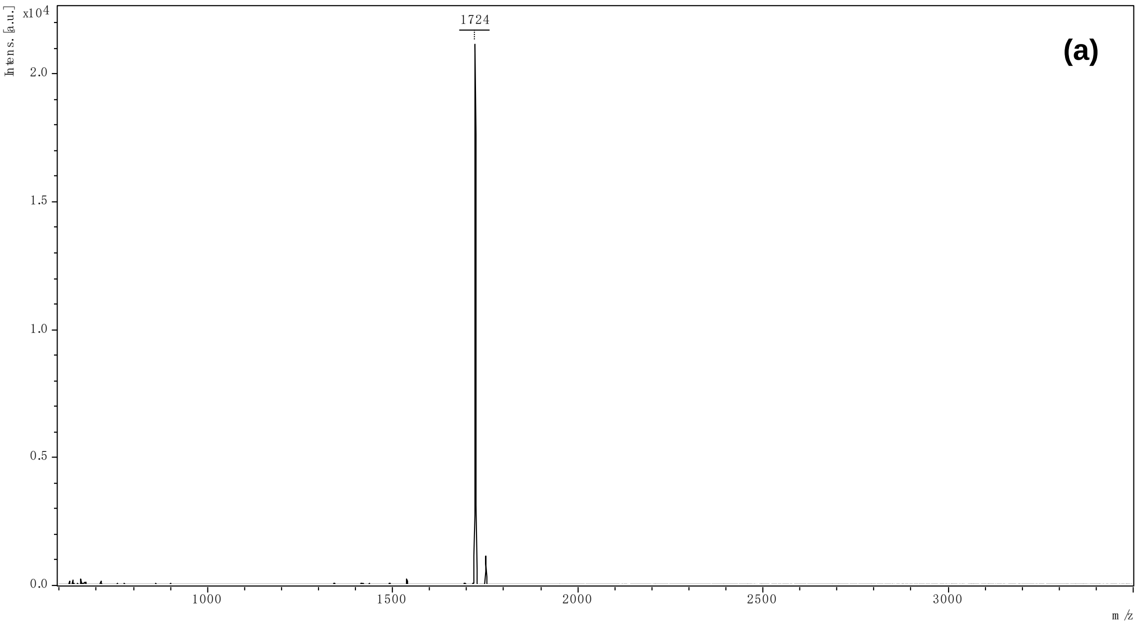

(a)
1724
x10^4
Intens. [a.u.]
2.0
1.5
1.0
0.5
0.0
1000
1500
2000
2500
3000
m/z

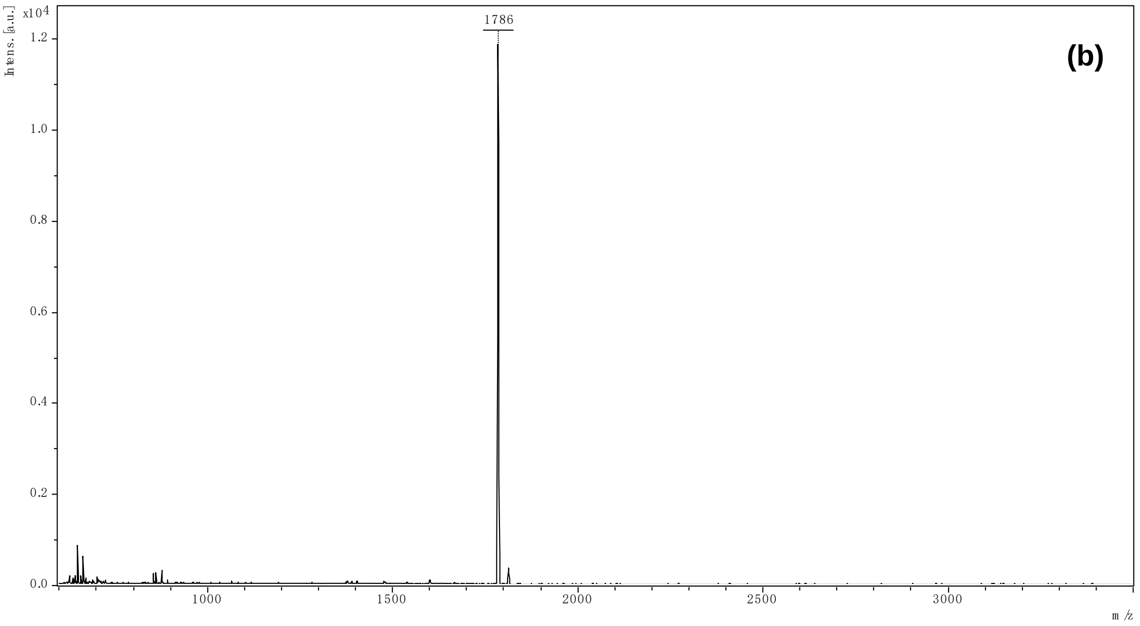

(b)
1786
x10^4
Intens. [a.u.]
1.2
1.0
0.8
0.6
0.4
0.2
0.0
1000
1500
2000
2500
3000
m/z

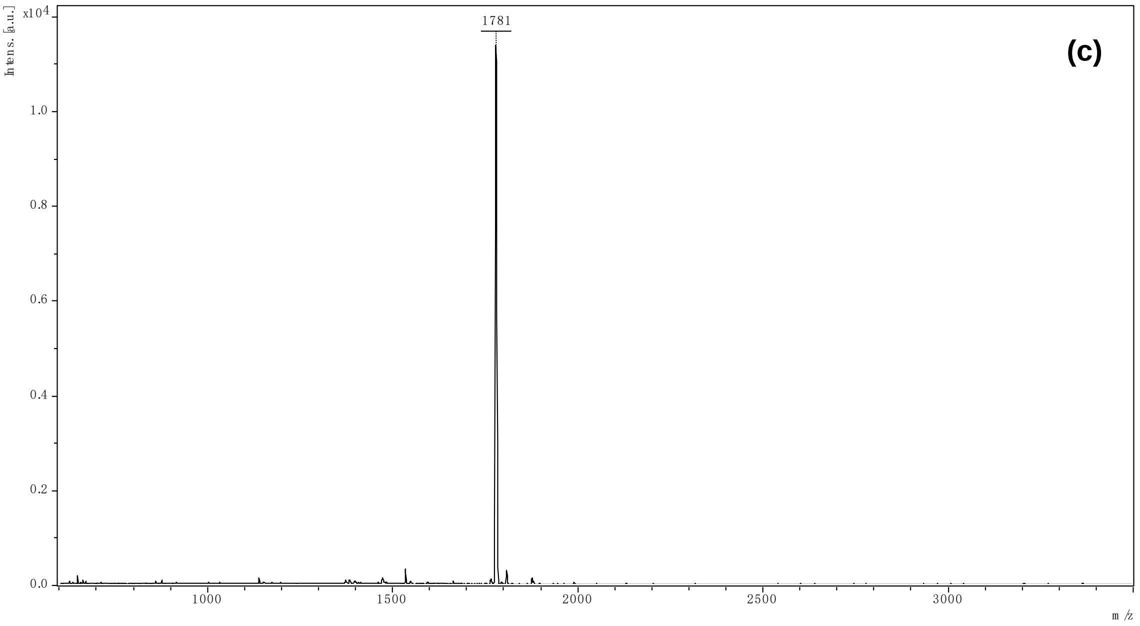

x10^4
Intens. [a.u.]
1781
(c)
1.0
0.8
0.6
0.4
0.2
0.0
1000
1500
2000
2500
3000
m/z

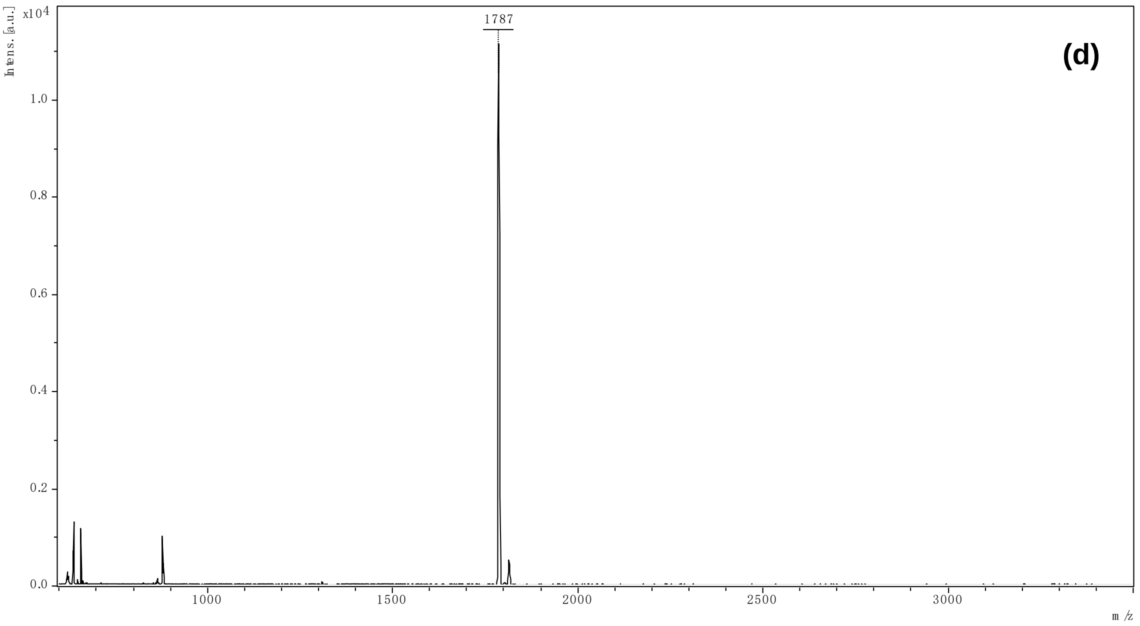

x10^4
Intens. [a.u.]
1787
(d)
1.0
0.8
0.6
0.4
0.2
0.0
1000
1500
2000
2500
3000
m/z

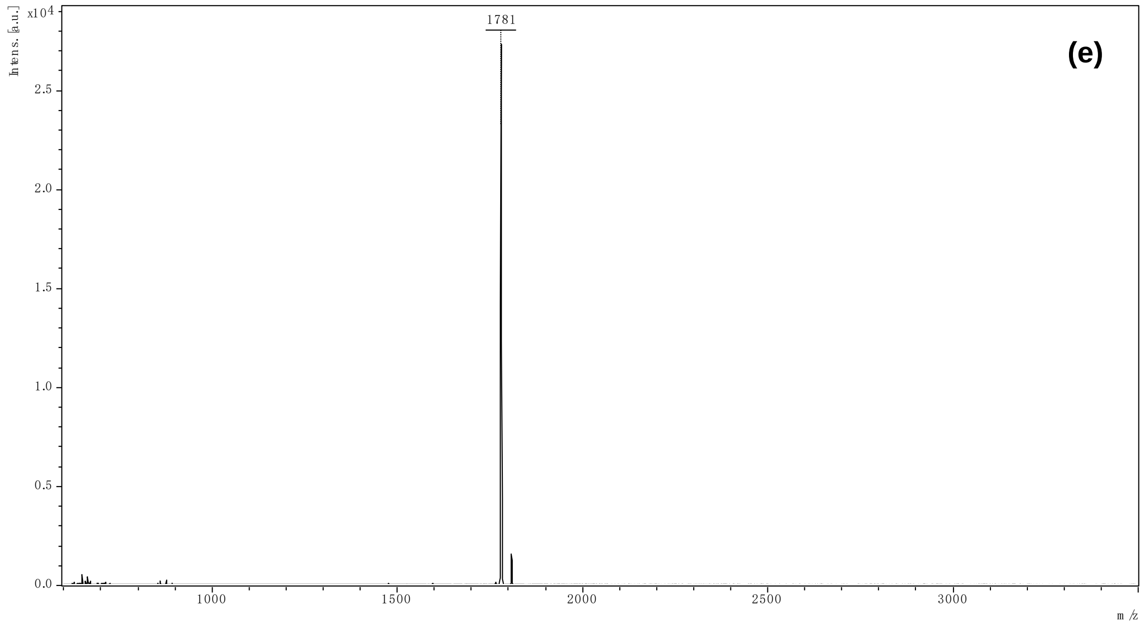

Figure 1. ^{13}C NMR (300 MHz, $CDCl_3$) of the (a) Pc, (b) CoPc, (c) CuPc, (d) NiPc and (e) ZnPc.
